# ALS-Linked P56S-VAPB Mutation Impairs the Formation of Multinuclear Myotube in C2C12 Cells

**DOI:** 10.3390/ijms160818628

**Published:** 2015-08-10

**Authors:** Yukako Tokutake, Keita Yamada, Masaki Ohata, Yoshihito Obayashi, Megumi Tsuchiya, Shinichi Yonekura

**Affiliations:** 1 Interdisciplinary Graduate School of Science and Technology, Shinshu University, 8304 Minamiminowa, Kamiina, Nagano 399-4598, Japan; E-Mail: 14st505j@shinshu-u.ac.jp; 2 Graduate School of Agriculture, Shinshu University, 8304 Minamiminowa, Kamiina, Nagano 399-4598, Japan.; E-Mails: 14aa114c@shinshu-u.ac.jp (K.Y.); 14aa104f@shinshu-u.ac.jp (M.O.); 15aa103a@shinshu-u.ac.jp (Y.O.); 15aa111b@shinshu-u.ac.jp (M.T.); 3 Department of Interdisciplinary Genome Sciences and Cell Metabolism, Institute for Biomedical Sciences, Interdisciplinary Cluster for Cutting Edge Research (ICCER), Shinshu University, 8304 Minamiminowa, Kamiina, Nagano 399-4598, Japan

**Keywords:** amyotrophic lateral sclerosis, satellite cells, vesicle-associated membrane protein associated protein B, differentiation, unfolded protein response

## Abstract

Amyotrophic lateral sclerosis (ALS) is a rapidly progressive neurodegenerative disorder that affects upper and lower motor neurons. Since motor neurons target skeletal muscles, the maintenance system of muscles is disturbed in ALS; however, the mechanism by which this occurs is unknown. In the present study, we investigated the effects of ALS-associated P56S-vesicle-associated membrane protein-associated protein B (VAPB) (P56S-VAPB) on the IRE1-XBP1 pathway, which is involved in the unfolded protein response (UPR) of the mouse myoblast cell line (C2C12 cells). Experiments with C2C12 cells transfected with wild-type wt-VAPB and P56S-VAPB expression vectors showed reduced myotube formation and aberrant myonuclear position in cells expressing P56S-VAPB. Activity of the IRE1-XBP1 pathway in the cells visualized with the ERAI system revealed that the pathway was disrupted in cells expressing P56S-VAPB, whereas the IRE1-XBP1 pathway activity was enhanced in the differentiation process of normal C2C12 cells. These results suggest that disruption of the IRE1-XBP1 pathway is a cause for the reduced myotube formation in P56S-VAPB-expressing cells. The expression level of the VAPB protein has been reported to be reduced in the neurons of patients with ALS. Therefore, it is expected that the IRE1-XBP1 pathway is also impaired in muscle tissues of patients with ALS, which causes a disturbance in the muscle maintenance system.

## 1. Introduction

Amyotrophic lateral sclerosis (ALS), the most common motor neuron disease in adults, is a neurodegenerative disease that involves the selective and systematic death of upper and lower motor neurons [1]. The death of upper motor neurons results in spasticity, and the degeneration of lower motor neurons cause muscle weakness and atrophy, followed by progressive paralysis [2]. Initially, ALS was thought to be a disease that primarily affects motor neurons, thereby causing muscle atrophy and paralysis. However, recent data suggest that non-neural cells, such as microglia, astrocytes, and skeletal muscle [3,4,5], could play a role in triggering motor neuron degeneration, and this evidence indicates that motor neuron death is, at least in part, non-cell autonomous.

Adult skeletal muscle can regenerate in response to exercise, injury, and disease. Skeletal muscle stem cells (satellite cells) are essential for skeletal muscle regeneration [6]. Satellite cells can differentiate and fuse to augment existing muscle fibers and to form new fibers. Quiescent satellite cells express the paired box transcription factor family member Pax7 [7]. During acute injury or muscle denervation, satellite cells are activated and they re-enter the cell cycle and begin proliferating to co-express myogenic differentiation antigen 1 (MyoD) [8,9]. Subsequently, satellite cells promote myogenin expression and elongate to form new myotubes to regenerate muscle [10]. Since skeletal muscle is the main target of motor neurons, muscle maintenance could be disturbed with motor neuron death. A recent study showed that ALS-derived satellite cells had an abnormal senescent-like morphology and were unable to completely differentiate [11]; however, the mechanisms are unclear.

Although most cases of ALS are sporadic, 5%–10% of cases are inherited in a dominant manner and are referred to as familial ALS [2]. A mutation in the gene encoding vesicle-associated membrane protein-associated protein B (VAPB) is associated with ALS type-8 (ALS8) [12]. Patients with ALS8 are linked to a mutation in the conserved major sperm protein homology domain, in which a proline is substituted by a serine (P56S-VAPB). VAPB protein is a type II integral membrane protein that mainly locates to the endoplasmic reticulum (ER). It contains a C-terminal transmembrane domain through which it is anchored in the ER membrane; the N-terminus of VAPB projects from the ER into the cytoplasm [13,14,15]. VAPB has been implicated in a variety of processes, including ER stress and the unfolded protein response (UPR) [16,17]. VAPB induces activation of one of the main UPR pathways, the IRE1-XBP1 pathway. ER stress is linked to the pathogenesis of ALS [18,19], and several studies suggest that the P56S-VAPB mutation is involved in an abnormal UPR; however, the mechanisms are unclear.

C2C12 cells, a mouse myoblast satellite cell line [20], have been extensively used as an *in vitro* model for studying the differentiation and regeneration of skeletal muscle [21]. We investigated the effects of the P56S mutation on myotube formation and the IRE1-XBP1 pathway in C2C12 cells. Here, we report for the first time that P56S-VAPB disrupted the formation of multinuclear myotubes. Furthermore, we found that the IRE1-XBP1 pathway was disrupted in P56S-VAPB-expressing C2C12 cells. These results suggest that P56S-VAPB disrupts the formation of multinuclear myotubes by suppressing the IRE1-XBP1 pathway. Our results may provide an explanation for the disturbed muscular maintenance system in patients with ALS.

## 2. Results and Discussion

### 2.1. P56S Mutation Results in Aberrant Aggregation of VAPB in C2C12 Cells

**Figure 1:**
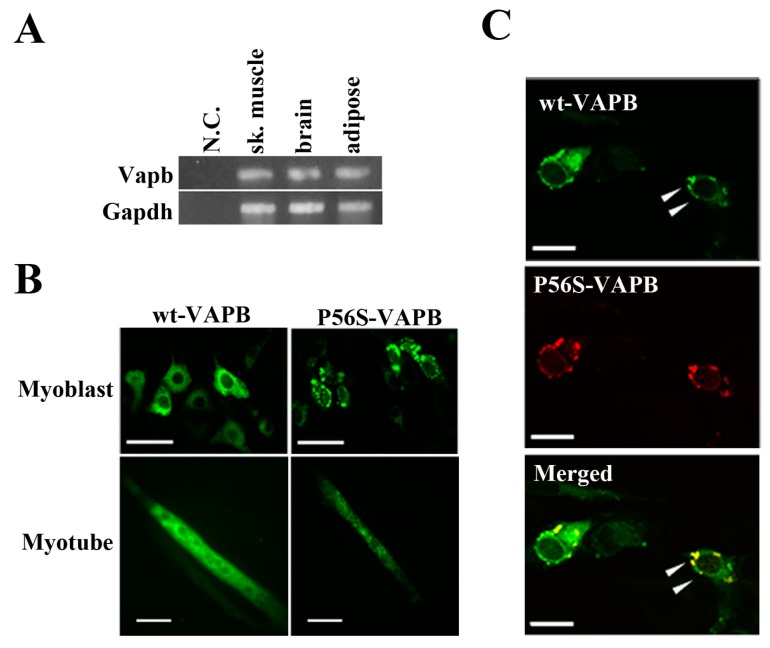
P56S mutation leads to aberrant aggregation of VAPB in C2C12 cells. (**A**) VAPB mRNA expression was analyzed by RT-PCR. Total RNA was collected from the indicated tissues of mice. N.C. indicates negative control (sample in which no reverse transcriptase was added); (**B**) C2C12 cells were transfected with the indicated plasmids and fixed either before inducing differentiation (**upper**: Myoblast) or five days after differentiation (**lower**: Myotube). The distribution of the VAPB protein is indicated by GFP expression; (**C**) C2C12 cells were co-transfected with vectors encoding C-terminally GFP-fused wt-VAPB and Ds-Red-fused P56S-VAPB, followed by fixation 24 h after transfection. Scale bar = 20 μm. The merged image is shown on the bottom. Examples of co-localization are indicated with arrowheads. These images are representative of three similar experiments.

First, we confirmed by RT-PCR experiments that VAPB mRNA was expressed in mouse skeletal muscle, brain, and adipose tissue (Figure 1A). Further, we transfected expression vectors carrying a gene for either wt-VAPB or P56S-VAPB with GFP added at the C-terminus into the C2C12 cells and observed the intracellular localization of these proteins. wt-VAPB was distributed uniformly in the cells, whereas P56S-VAPB aggregated in the cells (Figure 1B). Moreover, abnormal aggregation of P56S-VAPB was observed not only in undifferentiated cells, but also in myotubes, on the 6th day after the induction of differentiation. Furthermore, we co-transfected GFP-fused wt-VAPB and Ds-Red-fused P56S-VAPB genes into C2C12 cells, and we found that wt-VAPB and P56S-VAPB were co-localized as aggregates in the cells (Figure 1C).

### 2.2. P56S Mutation Reduced the Myotube Elongation and Results in Aberrant Localization Pattern of Myonuclei

**Figure 2:**
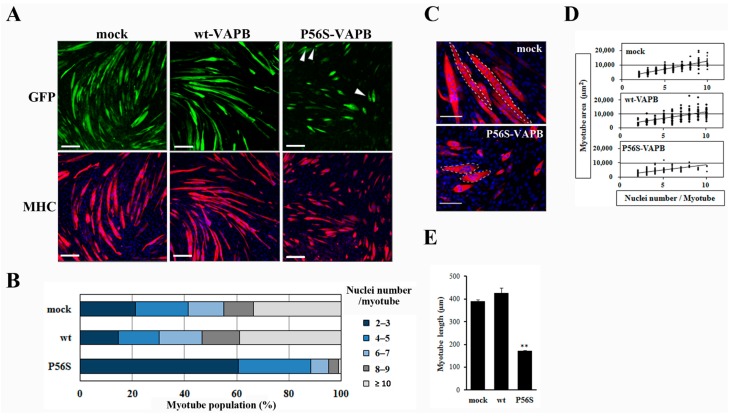
P56S mutation reduces myotube elongation and leads to an aberrant localization pattern of myonuclei. (**A**) C2C12 cells were transfected with the vectors encoding GFP (mock), GFP-fused wt-VAPB, or GFP-fused P56S-VAPB. Then, stable cell lines were generated by G418 selection. Stable cell lines were induced to differentiate until day five. Cells were immunostained using an anti-muscle heavy chain (MHC) antibody and secondary antibody conjugated to Alexa Fluor 568 (red). Nuclei were stained with DAPI (blue). Examples of P56S-VAPB aggregates are indicated with arrowheads. Scale bars = 200 µm; (**B**) The myotube population was segmented according to the number of nuclei per myotube. Each stable cell line was induced to differentiate until day five, and the number of nuclei contained in each myotube was counted. One hundred myotubes were included in each group; (**C**) Image comparison of the mock and P56S-VAPB expressing myotubes at 5 days after differentiation. The white dotted lines outline single myotubes. Mock myotubes contain six (**left**) and eight nuclei (**right**) each. P56S myotubes contain eight (**top**) and seven nuclei (**bottom**) each. Scale bars = 100 µm; (**D**) The myotube area was plotted according to the number of nuclei per myotube. Each stable cell line was induced to differentiate until day five, and the single myotube areas were measured using ImageJ software (National Institutes of Health, Bethesda, MD, USA). *n* = 214 (mock myotubes), 211 (wt-VAPB myotubes), and 300 (P56S-VAPB myotubes); (**E**) Average myotube length. Each stable line was induced to differentiate until day five, and the single myotube length was measured using ImageJ software (National Institutes of Health, Bethesda, MD, USA). The values are the means ± standard deviation for 300 myotubes in each group. ** *p* < 0.01 compared with mock.

Next, we prepared cell lines stably expressing wt-VAPB, P56S-VAPB, or GFP (mock) to examine the influences of P56S mutation on differentiation of the skeletal muscles. Immunofluorescent staining of the cells with anti-myosin heavy chain (MHC) antibodies on the 5th day after the induction of differentiation showed that myotube formation was suppressed in the P56S-VAPB-expressing cell line, whereas no appreciable difference was found between the GFP-expressing cell line and the wt-VAPB-expressing cell line (Figure 2A). The other P56S-VAPB-expressing cell line also showed reduced myotube formation (Supplementary Figure S1). Nuclei were counted in each myotube, and the proportions of myotubes containing different numbers of nuclei were analyzed. For the P56S-VAPB-expressing cell line, we found that myotubes containing two or three nuclei accounted for 60.7% of the entire set of myotubes analyzed, myotubes containing six or more nuclei accounted for a smaller proportion than in the other cell lines, and myotubes containing more than 10 nuclei were not formed (Figure 2B). The myotubes containing six or more nuclei in the P56S-VAPB-expressing cell line showed an aberrant localization pattern of nuclei and were smaller in size than the corresponding myotube subpopulations of other cell lines (Figure 2C). An analysis of the relationship between the number of nuclei and the myotube area showed that the area of the myotube was smaller in the P56S-VABP-expressing cell line than in other cell lines, even when myotubes containing the same number of nuclei were compared across the cell lines (Figure 2D). We next measured the myotube length of three cell lines. Quantitative analysis showed that myotube length of the P56S-VAPB-expressing cell line was significantly shorter than those of other cell lines (Figure 2E). We did not observe any reduction of the numbers of P56S-VAPB-expressing cells during myogenic differentiation (Supplementary Figure S2). The reduction of myotube formation was not correlated with any cell death. These results revealed that P56S-VAPB suppressed fusion and enlargement of C2C12 cells.

### 2.3. P56S Mutation Suppressed Fusion of C2C12 Cells for Late-Stage Differentiation

**Figure 3:**
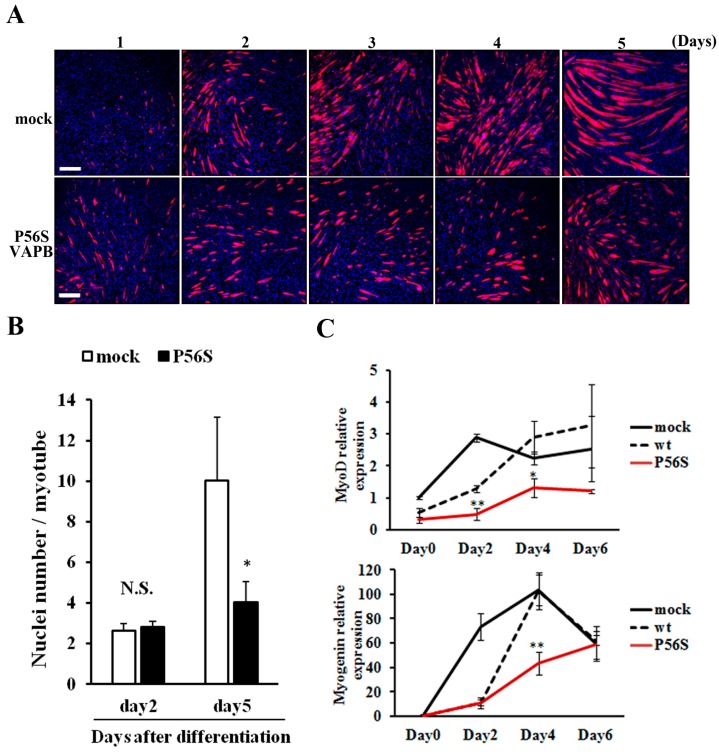
P56S mutation suppressed fusion of C2C12 cells for late-stage differentiation. (**A**) P56S mutation suppressed fusion of C2C12 cells for late-stage differentiation. GFP (mock) or P56S-VAPB stable expressing cell lines were induced to differentiate and detected MHC expression by immunostaining (red) on days 1–5 after differentiation. Nuclei were stained with DAPI (blue). Scale bars = 200 μm; (**B**) GFP (mock) or P56S-VAPB stable expressing cell lines were treated with differentiation medium and the number of nuclei per single myotube were counted at the indicated days after differentiation. The results are shown as means ± SEM (*n* = 3). * *p* < 0.05 compared with mock; (**C**) The mRNA expression of MyoD and Myogenin in GFP (mock), wt-VAPB-, or P56S-VAPB-expressing cells during differentiation induction. The level of mRNA was determined by real-time PCR and normalized to GAPDH. The results are expressed as means ± SEM for three independent experiments. * *p* < 0.05, ** *p* < 0.01 compared with mock and wt.

We compared changes in myotube formation over time between GFP-expressing and P56S-VAPB-expressing cell lines. No difference was observed between the two cell lines in the degree of myotube formation up to two days after differentiation induction; the difference appeared on the 3rd day and increased thereafter (Figure 3A). Regarding the number of nuclei contained in a myotube, the two cell lines showed no difference on the 2nd day after differentiation induction, but on 5th day, myotubes of the P56S-VAPB-expressing cell line contained significantly fewer nuclei than those of the GFP-expressing cell line (Figure 3B). We then analyzed the expression levels of myodifferentiation-related factors (MyoD and myogenin) over time in both cell lines using qRT-PCR. The results showed that the gene expression levels in the P56S-VAPB-expressing cell line were significantly lower than those in the GFP- and wt-VAPB-expressing cell line (Figure 3C), indicating that gene expression of myogenic differentiation-related factors in the process of differentiation is markedly suppressed in P56S-VAPB-expressing cells.

### 2.4. P56S Mutation Disrupted IRE1-XBP1 Pathway in C2C12 Cells

**Figure 4:**
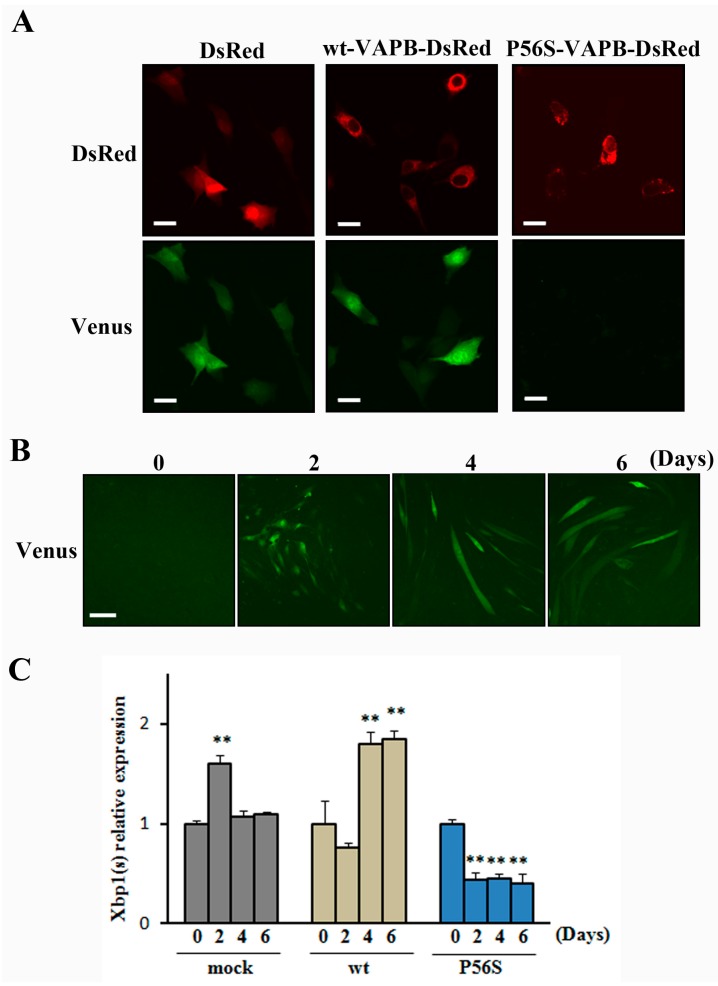
P56S mutation disrupted the IRE1-XBP1 pathway in C2C12 cells. (**A**) C2C12 cells co-expressed DsRed (mock), DsRed-fused wt-VAPB, DsRed-fused P56S-VAPB, and the ERAI gene (Venus). To induce ER stress, the cells were exposed to tunicamycin for 8 h and then visualized. Under ER stress conditions, IRE1 is activated, and splicing of XBP1 occurs, followed by expression of XBP1-Venus fluorescent fusion proteins. Scale bars = 20 µm; (**B**) C2C12 cells were transfected with the ERAI gene. XBP1-Venus fluorescence was detected as an indicator of IRE1 activation at the indicated days after differentiation. Scale bar = 100 μm; (**C**) The mRNA expression of XBP1(s) in GFP- (mock) or wt-VAPB- or P56S-VAPB-expressing cells during differentiation induction. The level of mRNA was determined by real-time PCR and normalized to GAPDH. The results are expressed as means ± SEM for three independent experiments. ** *p* < 0.01 compared with Day 0.

The IRE1-XBP1 pathway, which is involved in the UPR, has been reported to be disrupted in neuronal cells expressing P56S-VAPB [18,19]. Therefore, we studied whether the IRE1-XBP1 pathway was disrupted in C2C12 cells expressing P56S-VAPB. In this experiment, we used the ER stress Activated Indicator (ERAI) system capable of visualizing activities of the IRE1-XBP1 pathway. Under ER stress conditions, IRE1 is activated and splicing of XBP1 occurs, followed by expression of XBP1-Venus fluorescent fusion proteins. Observation of the cells treated with the ER stress inducer tunicamycin revealed that Venus fluorescence in P56S-VAPB-expressing cells was noticeably weaker than in DsRed-expressing cells or wt-VAPB-expressing cells (Figure 4A). In the absence of tunicamycin, we could not observe Venus fluorescence in all cells. (Supplementary Figure S3). This indicates that the IRE1-XBP1 pathway is disrupted in P56S-VAPB-expressing C2C12 cells. We then examined the activity state of the IRE1-XBP1 pathway over the differentiation process of C2C12 cells using the ERAI system, and we found that the activity was undetectable before differentiation but enhanced after differentiation (Figure 4B). Further, the mRNA expression level of XBP1(s) during the differentiation process was measured for GFP-, wt-VAPB-, and P56S-VAPB stable expressing cells using qRT-PCR. Although the expression level in GFP and wt-VAPB-expressing cells significantly increased with differentiation, no such increase was observed in P56S-VAPB-expressing cells (Figure 4C). Rather, we found decreased the expression level in P56S-VAPB-expressing cells. We confirmed the specificity of our primer set for the spliced form of XBP1 mRNA (Supplementary Figure S4).

### 2.5. Discussion

Previous studies on the motor neuron cell line NSC34 and the mouse adipocyte cell line 3T3-L1 have shown that P56S-VAPB aggregates in cells, whereas wt-VAPB distributes uniformly in cells [13,22]. In the present study, we co-transfected wt-VAPB and P56-VAPB into C2C12 cells, and found that these proteins were co-localized. This result is in agreement with the results of previous studies using NSC34 cells and 3T3-L1 cells [14,22], and suggests that P56S-VAPB recruits wt-VAPB, thereby inhibiting the function of wt-VAPB.

In the P56S-VAPB-expressing cells, myotube formation was suppressed, and nuclei localized in an abnormal manner in myotubes containing six or more nuclei. In addition, the number of nuclei contained in a myotube was not different between P56S-VAPB-expressing cells and the control cells on the 2nd day after differentiation induction, but was significantly lower in P56S-VAPB-expressing cells on the 5th day. In this experiment, we generated C-terminally GFP-fused wt-VAPB and P56S-VAPB, and examined the influences of P56S mutation on differentiation of the skeletal muscles. The extent of myotube formation is indistinguishable between GFP and GFP-fused wt-VAPB cells. As described above, the intracellular localization of both VAPB forms is consistent with previous studies. These results indicate that GFP at the C-terminus did not affect VAPB localization and function. Therefore, P56S mutation is caused for the reduced myotube formation. Aberrant localization of nuclei in myotubes has been reported to be found in amyotrophic diseases [23]. The amount of cytoplasm controlled by a single myonucleus is called the myonuclear domain. Each myonucleus governs a limited region in the cytoplasm and supplies proteins thereto [24]. Therefore, dysfunction of the myonuclear domain due to abnormal localization of nuclei is a likely cause of the suppressed myotube formation in P56S-VAPB-expressing cells.

Disruption to the IRE1-XBP1 pathway has been reported in P56S-VAPB-expressing neurons [18,19] and was also found in P56S-VAPB-expressing C2C12 cells in the present study. In addition, the IRE1-XBP1 pathway activity was enhanced in the differentiation process of normal C2C12 cells. Moreover, we found that the mRNA expression level of XBP1(s) was elevated in GFP and wt-VAPB-expressing cells after myogenic differentiation, but not in P56S-VAPB-expressing cells. IRE1 is an ER stress sensor, and when it is activated through accumulation of structurally abnormal proteins in the ER, the spliceosome-independent frame switch splicing is elicited and the active transcription factor XBP1(s) is produced from the spliced substrate XBP1 mRNA [25]. XBP1(s) contains both DNA-binding and transcription activation domains and induces gene transcription of ER chaperones and factors of the ER-associated protein degradation pathway [26]. In addition to the fact that the IRE1-XBP1 pathway plays a role in eliminating ER stress, XBP1 has been revealed to be an essential transcriptional factor for the terminal differentiation of B-cells into antibody-producing cells [27]. Recently, XBP1 has also been reported to be involved in the differentiation of adipocytes and osteoblasts [28,29]. Therefore, it is conceivable that the IRE1-XBP1 pathway is also involved in the differentiation of myocytes, which are derived from mesenchymal stem cells, as are adipocytes and osteoblasts. Although further detailed studies are required, the disrupted IRE1-XBP1 pathway is a plausible cause of the reduced myotube formation in P56S-VAPB-expressing cells.

Approximately 20% of familial ALS is considered to be attributable to SOD mutations. The VAPB protein has been reported to be expressed at a reduced level in the spinal cords of mutant SOD transgenic mice [14]. Moreover, the reduced VAPB protein expression has also been detected in the spinal cords of patients with sporadic ALS [30]. Hence, it is currently thought that the loss of VAPB protein function causes ALS. So far, there has been no report of the relationship between VAPB level and UPR. VAPB is seen to play a significant role in the UPR [16,17]. Recent study suggested that VAPB is required for ER protein quality control (ERQC). Loss of VAPB in flies showed various ERQC associated defects, including protein accumulation, ER expansion, and ER stress [31]. Although further investigations are required, loss of VAPB may lead to disruption of the UPR. Furthermore, previous report with the ALS mouse model suggested that XBP1(s) expression level was enhanced in SOD1^G93A^ motor neurons after adding thapsigargin, an inhibitor of the ER calcium pump, but the proportionate increase or slew rate of XBP1 activation after adding thapsigargin was higher in wild-type motor neurons [32]. It is speculated that regulation of UPR after additional ER stress induction is disrupted in ALS.

Dysfunctions of myosatellite cells from mutant SOD transgenic mice and patients with sporadic ALS have been reported [11,33], but the underlying mechanism remains unknown. Our present study revealed that P56S-VAPB disrupted the IRE1-XBP1 pathway in C2C12 cells and, presumably, this pathway is also disrupted in myosatellite cells of patients with other types of familial ALS and sporadic ALS. Future studies on the association between the IRE1-XBP1 pathway disruption and myonuclei that are aberrantly localized and those in myosatellite cells from patients with SOD mutations or sporadic ALS with a particular focus on the IRE1-XBP1 pathway are expected to result in the identification of the cause of the disturbed muscular maintenance system in patients with ALS.

## 3. Experimental Section

### 3.1. Reagents

Dulbecco’s modified Eagle medium (DMEM) was purchased from Gibco (Grand Island, NY, USA). Fetal bovine serum (FBS) was purchased from Microbiological Associates (Walkersville, MD, USA). Horse serum (HS) was purchased from Life Technologies (Carlsbad, CA, USA). All other compounds were from Wako Pure Chemical Company (Osaka, Japan).

### 3.2. Plasmids

pCAX-F-XBP1-ΔDBD-Venus (ERAI gene) [34] was kindly provided by Dr. Masayuki Miura (Tokyo University, Tokyo, Japan). Human cDNAs encoding VAPB (GenBankTM accession number NM_004738) were amplified as described previously [22]. P56S-VAPB was obtained by a QuikChange Site-Directed mutagenesis kit (Agilent Technologies, Santa Clara, CA, USA). wt-VAPB-GFP, P56S-VAPB-GFP, wt-VAPB-Ds-Red, and P56S-VAPB-Ds-Red were produced by cloning into pEGFP-C1 (Clontech, Cambridge, MA, USA) and pDs-Red-monomer-C1 (Clontech), respectively. The sequences of all PCR products were verified by sequencing.

### 3.3. Cell Culture and Transfection

C2C12 mouse myoblast cells were obtained from DS Pharma Biomedical (Osaka, Japan). C2C12 cells were grown in DMEM containing 10% FBS with penicillin and streptomycin (Sigma, Tokyo, Japan). At 80% confluence, myogenic differentiation was induced by switching to 2% HS-supplemented DMEM. Differentiation medium was changed every day during the course of myotube induction. All cultures were maintained at 37 °C under 5% CO_2_.

For the transient transfection, cells were transfected using Lipofectamine 2000 (Invitrogen, Carlsbad, CA, USA) according to the manufacturer’s protocol. To create stable cell lines, cells were transfected using the Lipofectamine 2000 reagent. After 48 h, cells were subjected to selection for stable integrants by exposure to 700 μg/mL G418 (Invitrogen) in complete medium containing 10% FBS. Selection was continued until monolayers were formed.

For the induction of pharmacological ER stress, C2C12 cells were exposed to growth medium containing 5 µg/mL tunicamycin (Sigma, Saint Louis, MO, USA) for 8 h.

For subcellular localization analysis, 6 h after transfection, cells were washed with phosphate-buffered saline (PBS) and fixed with 4% paraformaldehyde in PBS for 30 min. The cells were observed with a confocal laser-scanning microscope (FV1000; Olympus, Tokyo, Japan).

### 3.4. Immunocytochemistry

Cells were washed with PBS and fixed with 4% paraformaldehyde in PBS for 15 min. Following a block with 10% goat serum in PBS with 0.01% Triton-X100 (PBT), cells were incubated with a rabbit anti-skeletal myosin primary antibody (1:50) (Sigma) for 2 h at room temperature, and then washed three times in PBS. The cells were incubated Alexa-Fluor^®^ 568-conjugated goat anti-rabbit secondary antibody (1:200) IgG (H + L) (Life Technologies) for 1h and then washed five times. Nuclei were stained with DAPI (Life Technologies). The cells were observed with a confocal laser-scanning microscope (FLUOVIEW FV-1000, OLYMPUS).

### 3.5. Quantitative Analyses of Myotubes

C2C12 cells that had been immunostained for MHC and stained with DAPI were analyzed quantitatively. To analyze the number of nuclei and surface area per myotube, MHC-positive cells containing at least two nuclei were chosen and 100 MHC-positive cells per culture were included. Cell surface areas, cell numbers, and myotube lengths were analyzed using ImageJ software (National Institutes of Health, Bethesda, MD, USA) from the NIH.

### 3.6. Reverse Transcription (RT) Conventional PCR and Real-Time PCR

Total RNA was isolated from mouse tissue (for conventional PCR) and C2C12 cells (for real-time PCR) using the TRIzol reagent (Life Technologies, Carlsbad, CA, USA), according to the manufacturer’s instructions. For conventional PCR, samples were processed for cDNA synthesis using a ReverTra Ace^®^ (TOYOBO, Osaka, Japan). The amplification was performed with SapphireAmp Fast PCR Master Mix (TaKaRa Bio Inc., Shiga, Japan) using specific primers. Primers for VAPB and GAPDH were purchased from TaKaRa Bio Inc. (Shiga, Japan). For real-time PCR, samples were processed for cDNA synthesis using a qPCR RT Master Mix with gDNA Remover (TOYOBO, Japan). Quantitative real-time PCR was performed using SYBR Premix Ex TaqTM ll (TaKaRa Bio Inc., Shiga, Japan). Relative expression was quantified using the standard curve method and data were normalized to GAPDH gene expression. Primer sequences are as follows: Myogenin Forward: 5′-tacgtccatcgtggacagcat-3′, Reverse: 5′-tcagctaaattccctcgctgg-3′; MyoD Forward: 5′-tgagcaaagtgaatgaggccttc-3′, Reverse: 5′-tgcagaccttcgatgtagcggat-3′; XBP1(s) Forward: 5′-tgagaaccaggagttaagaacacg-3′, Reverse: 5′-cctgcacctgctgcggac-3′; Gapdh Forward: 5′-ttgtgatgggtgtgaaccacgag-3′, Reverse: 5′-catgagcccttccacaatgccaa-3′. GAPDH was used as an endogenous control. All quantitative RT-PCR was performed in triplicate on Thermal Cycler Dice^®^ Real Time System (TaKaRa Bio Inc.), based on a standard curve method. The sensitivity of the reaction and amplification of contaminating products, such as the extension of self-annealed primers, were evaluated by amplifying serial dilutions of cDNA. All data analysis was performed as recommended by the manufacturer.

### 3.7. Statistical Analysis

All data are represented as means ± standard error of the mean, with at least three repeats in each experimental group. Statistical significance was performed using the Student’s *t*-test. The test was considered significant at *p* < 0.05.

## 4. Conclusions

In summary, by analyzing the influences of P56S-VAPB on myotube formation and the IRE1-XBP1 pathway in C2C12 cells, P56S-VAPB was revealed to disrupt the formation of multinuclear myotubes by suppressing the IRE1-XBP1 pathway. As aberrant localization of nuclei in myotubes has been reported to be found in amyotrophic diseases, our result indicates that the P56S mutation leads to aberrant localization pattern of myonuclei. A previous report suggested that the reduced VAPB protein expression has also been detected in the spinal cords of patients with sporadic ALS. It is expected that future studies of the relationship between the disruption of the IRE1-XBP1 pathway and the aberrant localization of myonuclei using myosatellite cells of patients with sporadic ALS will identify the cause of the impaired muscular maintenance system in patients with ALS.

## Supplementary Materials

Supplementary materials can be found at http://www.mdpi.com/1422-0067/16/08/18628/s1.
