# Supplementary material for: ALS-Linked P56S-VAPB Mutation Impairs the Formation of Multinuclear Myotube in C2C12 Cells
Source: Int J Mol Sci. 2015 Aug 10;16(8):18628–41. doi: 10.3390/ijms160818628 (PMC4581263; doi:10.3390/ijms160818628)
Supplement: Supplementary file 1 [file ijms-16-18628-s001.pdf]

Supplementary Information

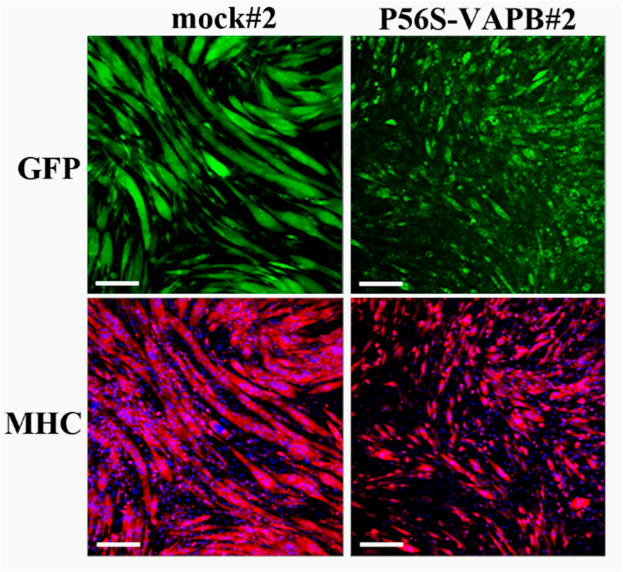

Figure S1. Immunofluorescent staining for MHC in the other P56S-VAPB stable expressing cell line. Stable cell lines were induced to differentiate until day five. Cells were immunostained using an anti-muscle heavy chain (MHC) antibody and secondary antibody conjugated to Alexa Fluor 568 (red). Nuclei were stained with DAPI (blue). Scale bars = 200 μm.

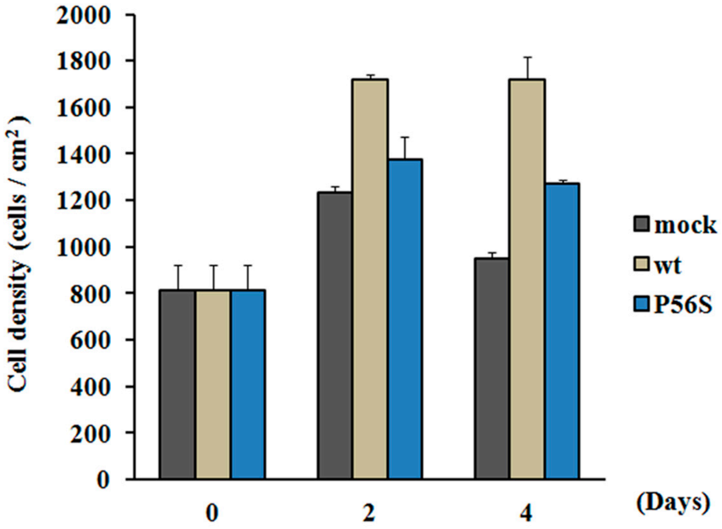

Figure S2. Time course of cell density following myogenic stimulation. Stable cell lines were induced to differentiate until day four. The numbers of cells was counted by labeling cell nuclei with DAPI using ImageJ software at the indicated days after differentiation.

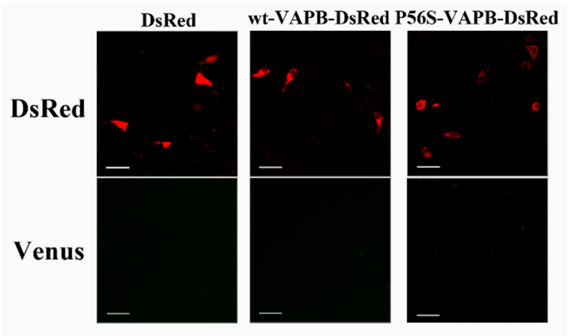

Figure S3. XBP1-venus is not detected in the absence of tunicamycin. C2C12 cells co-expressed DsRed (mock), DsRed-fused wt-VAPB, DsRed-fused P56S-VAPB, and the ERAI gene (Venus). The cells were exposed to PBS for 8 h and then visualized. Scale bar = 50 μm.

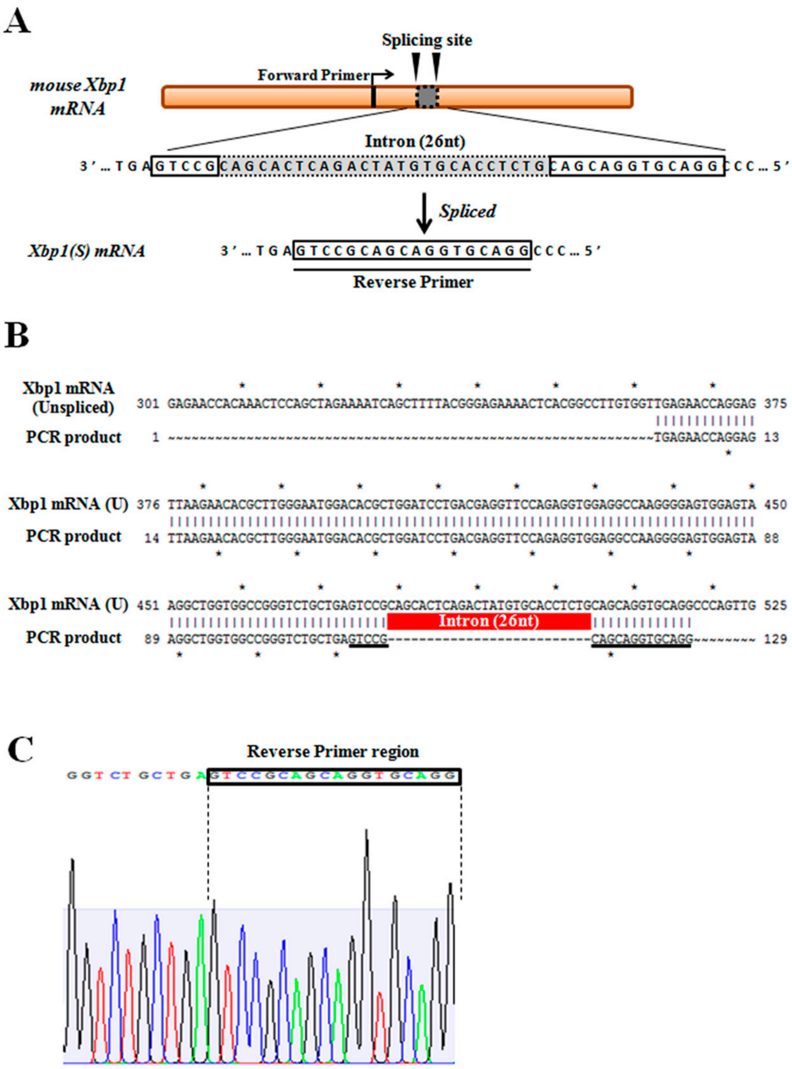

Figure S4. The specificity of primer set for the spliced form of XBP1 mRNA. (**A**) Scheme for analysis of the spliced form of XBP1 mRNA by qRT-PCR. The reverse primer spans the splice site; (**B**) Sequence comparison of the unspliced XBP1 and RT-PCR product. Bottom line indicates the reverse primer; (**C**) Sequence analysis of RT-PCR product. The sequence of reverse primer region is shown.
